# Symptoms of Dry Eye Disease in Hospitalized Patients with Coronavirus Disease 2019 (COVID-19)

**DOI:** 10.1155/2021/2678706

**Published:** 2021-12-08

**Authors:** Yan Wang, Sen Yang, Yue Zhang, Xin Zhang, Yaping Jiang, Xixi Wang, Pengxiang Zheng, Yihui Chen

**Affiliations:** ^1^Department of Neurology, Shanghai General Hospital, Shanghai Jiao Tong University School of Medicine, Shanghai, China; ^2^Department of General Practice, Yangpu Hospital, School of Medicine, Tongji University, Shanghai, China; ^3^Department of Bioinformatics and Biostatistics, School of Life Sciences and Biotechnology, Shanghai Jiao Tong University, Shanghai, China; ^4^Department of Ophthalmology, Yangpu Hospital, School of Medicine, Tongji University, Shanghai, China; ^5^Department of Cardiology, Yangpu Hospital, School of Medicine, Tongji University, Shanghai, China

## Abstract

**Background:**

We aimed to investigate the symptoms of the dry eye disease (DED) of hospitalized patients with coronavirus disease 2019 (COVID-19).

**Methods:**

This cross-sectional, observational study analysis included 91 hospitalized patients with confirmed COVID-19 in Wuhan, China. The Ocular Surface Disease Index (OSDI) and the five-item Dry Eye Questionnaire (DEQ-5) were used to assess the severity of DED symptoms in the patients, and the analysis of variance was used to determine the factors associated with DED.

**Results:**

A total of 42 patients consented to complete the investigation (response rate 46.15%). There were 26 (61.90%) patients who were diagnosed with DED symptoms by OSDI, and there were 28 (66.67%) patients with DED symptoms who were diagnosed by DEQ-5 score. For the biochemical tests, the patients with DED symptoms had lower aspartate aminotransferase (AST) levels compared to those with no DED symptoms (20.86 vs. 42.14, *p*=0.04). Further analysis showed that a previous history of cardiac or stroke disease (*p*=0.02) and typical symptoms of muscle soreness (*p*=0.03) were significantly different among the four DED symptoms groups on the basis of OSDI scores. The contributing factors of OSDI were mainly focused on visual function and environmental triggers.

**Conclusion:**

The incidence of DED symptoms is higher in hospitalized patients with COVID-19. The serum AST levels, history of cardiac or stroke disease, and the typical symptoms of muscle soreness may be the main impact factors on DED symptoms. We also need to pay more attention to the visual function and environmental triggers of hospitalized patients with COVID-19.

## 1. Introduction

At the end of 2019, coronavirus disease 2019 (COVID-19) broke out in Wuhan, China, and it has become a global pandemic caused by the highly transmissible severe acute respiratory syndrome coronavirus 2 (SARS-CoV-2) [1–4]. It caused considerable panic around the world because of its rapid transmission, high mortality, and changing virus variants [5].

Most patients with COVID-19 receive effective treatment through different measures. It has been reported that COVID-19 can affect the conjunctiva and cause acute viral conjunctivitis [6]. However, it is well known that, for most coronavirus infections, clinically significant conjunctivitis is rare, but there are some eye symptoms in hospitalized COVID-19 patients, such as eye dryness, blurred vision, eye soreness, and eye itch. Thus, we would like to determine the subclinical involvement of the anterior ocular surface. We reasoned that, in addition to direct viral infection, hospitalized patients may have eye discomfort related to dry eye disease (DED) because of the patients' long-term exposure to a negative-pressure chamber, relatively closed environment, heavy use of disinfectant in the environment, anxiety, and prolonged use of electronic products, along with other factors.

In this study, we investigated the subjective ocular symptoms of dry eye disease in a group of patients hospitalized with the confirmed diagnoses of COVID-19 in the epicenter of Wuhan.

## 2. Methods

### 2.1. Study Design and Participants

This is a cross-sectional, observational case series study at Leishenshan Hospital (Thunder God Mountain Hospital), a designated special medical center for COVID-19 in Wuhan, China. This work included two isolation wards as the domain of research from February 23, 2020, to April 5, 2020. The research protocol was approved by the Institutional Review Board of Yangpu Hospital and Leishenshan Hospital and was conducted in accordance with the tenets of the Declaration of Helsinki (No. LL-2020-KY-002).

The data of cases consistent with the diagnostic criteria of COVID-19 were collected and entered into the database by four designated and trained doctors (two ophthalmologists, one neurologist, and one physician), including sex, age, onset time, times of admission and discharge, previous history, laboratory findings, and pulmonary iconography data. Demographic, epidemiological, clinical, laboratory, and radiologic data were obtained from the patients' electronic medical records.

According to the World Health Organization (WHO) interim guidance [7], the diagnostic standards of a SARS-CoV-2 infection and COVID-19 are as follows: patients with severe acute respiratory infection symptoms (fever, cough, throat, chest ache, and cold), laboratory examination of the upper respiratory tract (nasopharyngeal and oropharyngeal) and lower respiratory tract (expectorated sputum, endotracheal aspirate, or bronchoalveolar lavage) for 2019-nCoV testing by reverse transcription-polymerase chain reaction (RT-PCR) with a positive result, and radiologic assessment of chest computed tomography (CT) revealing signs of viral pneumonia. The standards of cure and discharge included two consecutive negative results of the SARS-CoV-2 nucleic acid test (respiratory secretions samples, at least a 24-hour collection interval), the disappearance of clinical symptoms and signs, and a marked reduction of lung inflammation.

The serum alanine aminotransferase (ALT) (reference value 9–50 IU/L), aspartate aminotransferase (AST) (reference value 15–40 IU/L), and other laboratory examinations were measured by biochemical analysis. Cytokine measurements were detected by enzyme-linked immunosorbent assay (ELISA).

According to the WHO interim guidance and Diagnosis and Treatment Protocol for COVID-19 (Trial Version 7, China), there are four clinical types, which are classified as follows: (1) Mild type: the symptoms are mild, and no pneumonia was found on imaging; (2) Common type: clinical manifestations of fever, cough, sputum, as well as other symptoms, and pneumonia can be seen on imaging; (3) Severe type: respiratory rate >30 times/min; in the resting state, the oxygen saturation is less than 93% or arterial oxygen partial pressure (Pa02)/oxygen inhalation concentration (Fi02) < 300 mm Hg; pulmonary imaging showed that patients with obvious lesion progression >50% within 24-48 hours; (4) Critical type: respiratory failure occurs, mechanical ventilation is required, or shock or intensive care unit (ICU) monitoring and treatment are required for other organ failures. In this work, we targeted common and severe type patients as the study objects.

### 2.2. Symptomatic Dry Eye Disease Evaluation

Recruitment was conducted by two authors entering the isolation ward to consult with the patients. After explaining the purpose of the research and the research procedures and requirements, the hospitalized patients voluntarily completed the electronic questionnaires on a smartphone, which consisted of three parts: basic demographic and history of eye disorders, the Chinese versions of the Ocular Surface Disease Index (OSDI) [8], and the five-item Dry Eye Questionnaire (DEQ-5) [9].

The exclusion criteria were as follows: (1) the history of eye medication in the last 1 month; (2) active eye inflammation, such as acute and chronic dacryocystitis, acute and chronic dacryoadenitis, blepharitis, conjunctivitis, keratitis, sclerotitis, uveitis, and active fundus lesions in the last 3 months; (3) the history of contact lens wear in the last 3 months; (4) the history of eye trauma and surgery in the last 6 months; and (5) pterygium, glaucoma, hyperthyroidism, rheumatism, dry eye syndrome, cicatricial conjunctivitis, eyelid trichiasis, and other disorders affecting tear secretion.

The Dry Eye Questionnaire Evaluation was classified as follows: (1) OSDI: ≤12 (no DED symptoms), 13 ≤ d ≤ 22 (mild DED symptoms), 23 ≤ d ≤ 32 (moderate DED symptoms), and ≥33 (severe DED symptoms) and (2) DEQ-5: ≤5 (no DED symptoms) and ≥6 (DED symptoms).

### 2.3. Statistical Analyses

The measurement data are expressed as means ± standard derivations. The group means were compared by *t*-test or one-way analysis of variance (ANOVA). The categorical variables were analyzed by the chi-square test or Fisher's test. The contributing factors proportion of OSDI and DEQ-5 for subscale scores were expressed using a radar graph. The analysis of all data was performed using R3.5.0 for Windows 7.0; *p* < 0.05 was considered statistically significant.

## 3. Results

### 3.1. Demographic and Clinical Characteristics

Of a total of 91 hospitalized patients with confirmed COVID-19, 42 patients who consented to complete the investigation were ultimately included in the analysis (for a response rate of 46.15%).

Among the participants, the mean (standard deviation, SD) age was 55.83 (11.98) years. Of these, 17 were male (40.48%) and 25 were female (59.52%). These patients were classified into two types clinically: common type, 26 (61.90%), and severe type, 16 (38.10%).

There were 28 (66.67%) cases who had underlying diseases, including hypertension, 16 (38.10%); diabetes, 7 (16.67%); digestive system diseases, 7 (16.67%); respiratory disease, 5 (11.90%); cardiac or stroke disease, 3 (7.14%); and thyroid disease, 2 (4.76%).

The main clinical manifestations at the onset were fever, 33 (78.57%); cough, 28 (66.67%); fatigue, 16 (38.10%); anhelation, 14 (33.33%); muscle soreness, 12 (28.57%); bosom frowsty and/or ache, 6 (14.29%); pharyngalgia, 6 (14.29%); and abdominal pain and/or diarrhea, 5 (11.90%). Upon comparing the clinical characteristics and previous history, there were no differences between the patients with the common and severe types of disease (*p* > 0.05).

Moreover, according to the DED score valuation standards, the results of the OSDI showed that there were 16 (38.10%) cases with no DED symptoms and 26 (61.90%) cases with DED symptoms. The DEQ-5 showed that there were 14 (33.33%) cases with no DED symptoms and 28 (66.67%) cases with DED symptoms. Further analysis showed that there was no remarkable difference in the DED symptoms among COVID-19 patients with different clinical types (*p* > 0.05) (Table 1).

### 3.2. Laboratory Findings of COVID-19 Patients with and without DED Symptoms

As per the OSDI and DEQ-5 scores, there were no differences in the routine blood work, including hemoglobin, white blood cells, neutrophils, lymphocytes, monocytes, and platelets, between the groups with DED symptoms and no DED symptoms.

For the biochemical tests of the OSDI score, the patients with DED symptoms had lower AST levels when compared to those with no DED symptoms (20.86 *vs.* 42.14, *p*=0.04). Other indicators such as ALT, serum creatinine, serum urea nitrogen, creatine kinase, D-dimer, and procalcitonin (PCT) were not significantly different between the patients with and without the DED symptoms (*p* > 0.05).

The results of cytokine analysis showed that patients with DED symptoms had lower tumor necrosis factor-*α* (TNF-*α*) levels and interleukin-2 (IL-2) receptor levels when compared to those with no DED symptoms, with no significant difference (OSDI-TNF-*α* (400.46 *vs.* 452.09, *p*=0.71); DEQ-5-TNF-*α* (416.14 *vs.* 420.33, *p*=0.98) and OSDI-IL-2 receptor (6.59 *vs.* 7.01, *p*=0.67); DEQ-5-IL-2 receptor (6.48 *vs.* 7.17, *p*=0.47). Other cytokines such as interleukin-6 (IL-6), interleukin-8 (IL-8), interleukin-1*β* (IL-1*β*), and interleukin-10 (IL-10) had no clinical significance (Table 2).

### 3.3. Clinical Characteristics of COVID-19 Patients from Different Subtype DED Symptoms Score Groups

OSDI scores showed that 33.33% (*n* = 14) of the patients had no DED symptoms, 11.9% (*n* = 5) had mild DED symptoms, 38.10% (*n* = 16) had moderate DED symptoms, and 16.67% (*n* = 7) had severe DED symptoms.

Outside the previous history of cardiac or stroke disease (*p*=0.02) and typical symptoms of muscle soreness (*p*=0.03), which had significant differences, there were no significant differences among the four subtype groups based on the clinical characteristics of age, sex, hospitalization, history of eye diseases, other previous histories, and other typical COVID-19 symptoms (*p* > 0.05).

The DEQ-5 score showed that the ratios of female (*p*=0.51), hypertension (*p*=0.70), myopia (*p*=0.69), and typical COVID-19 symptoms of fever (*p*=0.27) and muscle soreness (*p*=0.09) were higher in the DED symptoms group than in the no-DED symptoms group, but these differences were not statistically significant (Table 3).

### 3.4. The Proportion of OSDI for Subscale Scores in COVID-19 Patients from Different Subtype DED Symptoms

**Figure 1:**
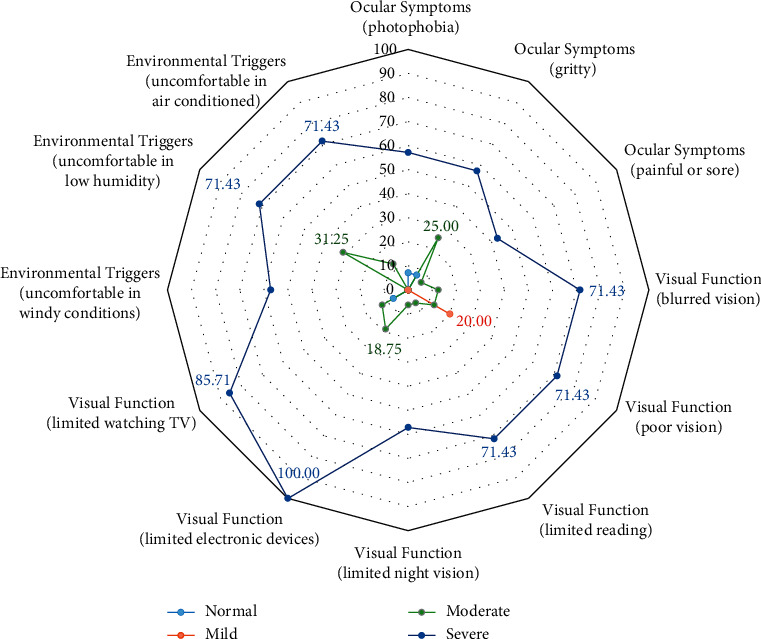
OSDI subscale scores in different subtype DED symptoms. The contributing factors of severe DED symptoms in hospitalized COVID-19 patients were visual function and environmental triggers. The contributing factors of moderate DED symptoms in hospitalized COVID-19 patients were environmental triggers (uncomfortable in low humidity), ocular symptoms (gritty), and visual function (limited electronic devices). In mild DED symptoms group, the contributing factor was mainly focused on visual function (poor vision).

The contributing factors of severe DED symptoms in hospitalized COVID-19 patients were visual function (limited electronic devices) (100.00%) and visual function (limited watching TV) (85.71%). The contributing factors of moderate DED symptoms in hospitalized COVID-19 patients were environmental triggers (uncomfortable in low humidity) (31.25%), ocular symptoms (gritty) (25.00%), and visual function (limited electronic devices) (18.75%). In the mild DED symptoms group, the contributing factor was mainly focused on visual function (poor vision) (20.00%) (Figure 1).

## 4. Discussion

The evidence in the latest study shows that SARS-CoV-2 can infect ocular surfaces, leading to excessive tearing and redness [10]. The clinical manifestations of conjunctivitis are easily confused with those of DED. However, in our study, the COVID-19 patients did not exhibit obvious ophthalmologic symptoms such as photophobia, lacrimation, mucous secretion, and conjunctival congestion on early infection. There are many common symptoms or similar symptoms between DED and viral conjunctivitis, and the conditions can even coexist [11]. However, there is still evidence of the need to screen for differences between DED and conjunctivitis. Thus, when COVID-19 patients present with DED, the clinical characteristics of occurrence, development, and prognosis of COVID-19 should be considered.

DED is considered a symptomatic disease because the rates of prevalence based on symptom-reporting are more consistent than those based on the signs. The population-based studies reporting the prevalence of DED based on the symptoms are heterogeneous. The prevalence of DED for studies involving the symptoms with or without the signs ranged from approximately 5% to 50% [12]. In Asian studies, the overall prevalence of DED symptoms reports ranged between 14.4 and 24.4% [13–15].

Studies have defined the DED symptoms using different methods, including symptom questionnaires and the frequency of symptoms (foreign body sensation, dryness, irritation, itching, or burning). Among the currently validated dry eye-specific questionnaires, the OSDI and DEQ-5 are the two instruments recommended by the Tear Film and Ocular Surface Society (TFOS) Dry Eye Workshop (DEWS) II diagnostic methodology report [16]. Symptom screening with the DEQ-5 or OSDI confirms that a patient might have DED and suggests conducting the diagnostic tests of signs, such as breakup time, Schirmer test, and ocular surface staining with fluorescein. The OSDI, including 12 questions, measures ocular symptoms, environmental triggers, vision-related functions, and limitations. The DEQ-5, consisting of 5 short questions, is sensitive to dry eye severity. Our study showed a relatively high prevalence rates of DED symptoms of 61.9% based on an OSDI questionnaire score above 12 and 66.67% based on a DEQ-5 score above 5. We also found that the proportions of DED symptoms were not significantly different between the two COVID-19 clinical types (common and severe). These results suggest that the DED symptoms had a high incidence in COVID-19 patients, with no relationship with the severity of COVID-19.

Thus, what factors leading to DED showed a high morbidity rate with COVID-19 patients? In evaluating the risk factors of DED, environmental exposures, electronic products use, diet and nutritional factors, affective and somatoform disorders, lifestyle factors, anxiety, chronic pain, depression, sleep disorder, and blepharitis-like changes (caused by SARS-CoV-2 infection) were identified and considered. Blepharitis is an extremely frequent cause of dry eye disease. Patients with COVID-19 have blepharitis-like ocular changes, primarily because of SARS-CoV-2 ocular tropism [17]. When the virus invades the eye, the ocular surface is able to form a defensive barrier against a variety of pathogens by producing immunoglobulins, lysozyme, and other antimicrobial peptides. Therefore, the ocular evaluation of patients with COVID-19 will reveal a mild conjunctival congestion and increased secretions in some patients, along with a marginal eyelid congestion and other signs of blepharitis. These symptoms may be associated with SARS-CoV-2 activity in the ocular surface epithelium and glands, leading to tear film instability or tear reduction with DED symptoms such as dry eyes, burning, and foreign-body sensation [18]. An association between DED and several affective disorders, including anxiety and depression, is reported most frequently [19, 20]. Clinical observations showed that COVID-19 patients had some psychological problems, such as nervousness, anxiety, depression, and dread, which may act as triggers to alter the immune response and consequently increase the probability of DED [21]. DED could affect the vision quality through tear-related changes. Likewise, an impaired visual performance can lead to some psychological problems, such as depression, anxiety, and chronic pain, which could negatively affect the patients' daily activities, work, and physical rehabilitation. It is a vicious circle that requires some medical intervention. In addition, several environmental factors, such as air pollution, wind, low humidity, and high altitude, are suggested to impact DED [22–25]. In our study, all COVID-19 patients were in separate negative-pressure wards with independent air-conditioning environments. It is known that there is a high prevalence of dry eye symptoms among video display terminal (VDT) users, and it has been hypothesized that, during visual display use, a diminished blink frequency rate contributes to an accelerated tear evaporation, leading to tear film instability, mild epithelial damage, and dry eye symptoms [26]. COVID-19 hospitalized patients had more free time to use VDTs, such as smartphones, PADs, and TV. In our opinion, this is a major factor for DED symptoms with COVID-19 hospitalized patients.

Moreover, we analyzed the relationship of laboratory findings in patients between the no DED symptom and DED symptom groups. Routine blood, ALT, serum creatinine, serum urea nitrogen, creatine kinase, D-Dimer, PCT, and serum cytokine levels of COVID-19 patients were not related to DED, but serum AST levels might be related. The latest studies have revealed that the patients with DED symptoms had higher tear levels of (IL)-1*β*, IL-6, IL-8, IL-10, and TNF-*α* than those with no DED symptoms [27]. However, our study did not reveal the same tendencies with serum cytokines. The findings demonstrate the differences in the expression between the serum and tear samples and put forward that the patients with DED had lower serum AST levels, which has not yet been reported. Thus, we need further research to understand the potential clinical characteristics.

The accepted risk factors linked to DED include age, female sex, smoking, diabetes, vitamin deficiency, diabetes, keratoplasty, alcohol, and anticholinergic drugs [28]. For our data, there were no significant variables related to DED, including age, sex, hospitalization, and a history of eye diseases. However, DED may be related to a previous history of cardiac or stroke disease and typical COVID-19 symptoms such as muscle soreness. The reason for this relationship may be that SARS-CoV-2 infects the skeletal muscle cells, which mediates an immune response in the tissue. However, more clinical evidence will be needed to determine the association of the same pathophysiological mechanisms on the lacrimal gland.

In this study, we analyzed three subscales of OSDI, i.e., the ocular symptoms, vision-related function, and environmental triggers. It showed that the contributing factors of DED symptoms in hospitalized COVID-19 patients were mainly focused on vision-related function and environmental triggers. This result indicates that the overuse of electronic terminals and environmental triggers may play an important role in the development of symptomatic dry eye disease in hospitalized patients of COVID-19.

DED affects both vision and comfort of the eye. Fortunately, in our study, the majority of patients with the symptoms of eye strain, eye fatigue, burning, irritation, redness, and blurred vision have received health guidance (including advice to shorten VDT time, proper eye massage, and relaxation). In Oliverio's study, povidone-iodine (PVI) was found to be a broad antibacterial with good efficacy and tolerability against drug-resistant microorganisms, but higher concentrations tend to cause corneal cell death and persistent epithelial defects. However, lower concentrations are commonly used clinically to prevent intraocular surgery and for the validation of the ocular surface. A low concentration of PVI at 0.6% has a significant effect in improving the symptoms of dry eye disease [29]. These measures have been proved to alleviate the symptoms of DED.

There are some limitations to this study. This article analyzed the various factors of common clinical manifestations and their relationship with DED, but the evidence was still insufficient.

On the one hand, only 42 patients were included in our study, which could cause relativity biases; on the other hand, because of the condition limitations, the COVID-19 patients had no opportunity to complete the laboratory examinations of DED, such as tear volume and tear stability tests. Additionally, there is some limitation to the diagnosis of DED only via DED questionnaire scores.

In summary, our research has suggested a possibility that the DED symptoms are more prevalent in the population of hospitalized patients with COVID-19. The serum AST levels, history of cardiac or stroke disease, and typical symptoms of muscle soreness may be the impact factors on DED symptoms. However, it is hard to find the inner relationship between them based on our limited data. Further research is required to understand the potential clinical characteristics. Meanwhile, we also need to pay more attention to the visual function and environmental triggers of hospitalized patients with COVID-19.

## Figures and Tables

**Table 1:** The characteristics of patients with COVID-19.

Characteristic	No. (%)			*P* value
	Total (*N* = 42)	Common (*N* = 26, 61.90%)	Severe (*N* = 16, 38.10%)	
Age (SD), years	55.83 (11.98)	56.31 (11.48)	55.06 (13.09)	0.75
Age, y				
<50	15 (35.71)	8 (30.77)	7 (43.75)	0.60
≥50	27 (64.29)	18 (69.23)	9 (56.25)	
Gender				
Female	25 (59.52)	19 (73.08)	6 (37.50)	0.05
Male	17 (40.48)	7 (26.92)	10 (62.50)	
Hospitalization (SD), days	30.12 (7.31)	30.12 (7.31)	30.12 (7.48)	0.10
Previous history				
Hypertension	16 (38.10)	11 (42.31)	5 (31.25)	0.70
Diabetes	7 (16.67)	5 (19.23)	2 (12.5)	0.70
Cardiac or stroke disease	3 (7.14)	2 (7.69)	1 (6.25)	1.00
Thyroid disease	2 (4.76)	1 (3.85)	1 (6.25)	1.00
Kidney disease	1 (2.38)	1 (3.85)	0 (0)	1.00
Digestive system disease	7 (16.67)	4 (15.38)	3 (18.75)	1.00
Respiratory system disease	5 (11.90)	3 (11.54)	2 (12.5)	1.00
History of eye diseases				
Glaucoma	2 (4.76)	2(7.69)	0 (0.00)	0.57
Cataract	3 (7.14)	2 (7.69)	1 (6.25)	1.00
Myopia	8 (19.05)	8 (30.77)	0 (0.00)	0.02
Ocular fundus disease	1 (2.38)	1 (3.85)	0 (0.00)	1.00
Ophthalmic medication (within one month)	8 (19.05)	8 (30.77)	0 (0.00)	0.02
Activity of ophthalmic disease (within three months)	2 (4.76)	2 (7.69)	0 (0.00)	0.52
Ocular operation or trauma (within three months)	1 (2.38)	1 (3.85)	0 (0.00)	1.00
Typical symptoms				
Fever	33 (78.57)	18 (69.23)	15 (93.75)	0.12
Cough	28 (66.67)	17 (65.38)	11 (68.75)	1.00
Pharyngodynia	6 (14.29)	4 (15.38)	2 (12.50)	1.00
Anhelation	14 (33.33)	8 (30.77)	6 (37.50)	0.74
Fatigue	16 (38.10)	11 (42.31)	5 (31.25)	0.53
Muscle soreness	12 (28.57)	7 (26.92)	5 (3125)	1.00
Bosom frowsty and (or) ache	6 (14.29)	3 (11.54)	3 (18.75)	0.66
Abdominal pain, diarrhea	5 (11.90)	3 (11.54)	2 (12.5)	1.00
OSDI				
≤12 (no DED)	16 (38.10)	12 (46.15)	4 (25.00)	0.21
≥13 (DED)	26 (61.90)	14 (53.85)	12 (75.00)	
DEQ-5				
≤5 (no DED)	14 (33.33)	11 (42.31)	3 (18.75)	0.18
≥6 (DED)	28 (66.67)	25 (57.69)	13 (81.25)	

**Table 2:** The laboratory findings of COVID-19 patients with DED symptoms.

Laboratory examination	Median (SD)					
	OSDI score			DEQ-5 score		
	NO DED (score ≤12) (*n* = 14, 33.33%)	DED (score≥13) (*n* = 28, 66.67%)	*P* value	NO DED (score≤5) (*n* = 16, 38.10%)	DED (score≥6) (*n* = 26, 61.9%)	*P* value
Blood routine (count, 10^9^/L)						
Hemoglobin	118.50 (17.64)	112.86 (18.37)	0.35	120.19 (15.78)	111.39 (18.92)	0.13
White blood cell	5.43 (1.60)	5.61 (1.67)	0.74	5.79 (1.21)	5.40 (1.85)	0.45
Neutrophil	3.49 (1.28)	3.20 (1.07)	0.44	3.66 (1.01)	3.07 (1.17)	0.11
Lymphocyte	1.44 (0.48)	1.62 (0.50)	0.28	1.49 (0.42)	1.60 (0.54)	0.50
Monocytes	0.45 (0.19)	0.52 (0.18)	0.25	0.46 (0.13)	0.52 (0.21)	0.34
Platelet	253.36 (140.27)	254.57 (81.92)	0.97	222.56 (80.09)	273.62 (112.24)	0.12
Biochemical tests						
ALT (IU/L)	49.50 (55.48)	27.21 (16.93)	0.06	41.81 (31.01)	30.23 (38.28)	0.31
AST (IU/L)	42.14 (48.45)	20.86 (14.26)	0.04	33.31 (42.12)	24.65 (22.71)	0.39
Serum creatinine (*μ*mmol/L)	67.86 (40.23)	61.06 (15.06)	0.43	70.43 (36.85)	58.96 (15.40)	0.17
Serum urea nitrogen (mmol/L)	4.57 (2.27)	4.59 (1.47)	0.98	4.29 (2.22)	4.76 (1.40)	0.40
Creatine kinase (IU/L)	45.54 (21.37)	48.43 (30.77)	0.76	42.800 (17.60)	50.23 (32.41)	0.42
D-Dimer (mg/L)	1.15 (1.56)	0.87 (1.24)	0.52	1.32 (2.00)	0.74 (0.65)	0.18
PCT (ng/L)	0.06 (0.08)	0.05 (0.08)	0.77	0.05 (0.04)	0.06 (0.09)	0.70
Serum cytokines						
TNF-*α*	452.09 (608.27)	400.46 (163.80)	0.71	420.33 (594.99)	416.14 (139.40)	0.98
IL-2 receptor	7.01 (4.21)	6.59 (1.26)	0.67	7.17 (4.05)	6.48 (1.18)	0.47

**Table 3:** The clinical characteristics of COVID-19 patients with subtype DED symptoms score groups.

Characteristic	No.(%)							
	OSDI score					DEQ-5 score		
	NO DED (*n* = 14, 33.33%)	Mild DED (*n* = 5, 11.90%)	Moderate DED (*n* = 16, 38.10%)	Severe DED (*n* = 7, 16.67%)	*P* value	NO DED (*n* = 16, 38.10%)	DED (*n* = 26, 61.9%)	*P* value
Age (SD), years	57.50 (14.60)	49.60 (6.80)	54.50 (11.91)	60.60 (8.33)	0.88	54.12 (13.57)	56.89 (11.04)	0.748
Age,y								
<50	4 (28.57)	3 (60.00)	7 (43.75)	1 (14.29)	0.34	7 (43.75)	8 (30.77)	0.60
≥50	10 (71.43)	2 (40.00)	9 (56.25)	6 (85.71)		9 (56.25)	18 (69.23)	
Gender								
Male	5 (35.71)	1 (20.00)	10 (62.55)	1 (14.29)	0.11	8 (50.00)	9 (34.62)	0.51
Female	9 (64.29)	4 (80.00)	6 (37.50)	6 (85.71)		8 (50.00)	17 (65.38)	
Hospitalization (SD),days	28.21 (6.59)	31.20 (2.39)	31.19 (8.02)	30.71 (9.69)	0.33	28.69 (6.84)	31.00 (7.58)	0.33
Previous history								
Hypertension	7 (50.00)	1 (20.00)	4 (25.00)	4 (57.14)	0.33	5 (31.25)	11 (42.31)	0.70
Diabetes	3 (21.42)	2 (40.00)	1 (6.25)	1 (14.29)	0.27	5 (31.25)	2 (7.69)	0.09
Cardiac or stroke disease	0 (0.00)	1 (20.00)	0 (0.00)	2 (28.57)	0.02	0 (0.00)	3 (11.54)	0.28
Thyroid disease	1 (7.14)	1 (20.00)	0 (0.00)	0 (0.00)	0.16	1 (6.25)	1 (3.85)	1.00
Kidney disease	1 (7.14)	0 (0.00)	0 (0.00)	0 (0.00)	0.62			
Digestive system disease	2 (14.29)	1 (20.00)	3 (18.75)	1 (14.29)	1.00	2 (12.50)	5 (19.23)	0.69
Respiratory system diseases	0 (0.00)	1 (20.00)	3 (18.75)	1 (14.29)	0.28	0 (0.00)	5 (19.23)	0.14
History of eye diseases								
Glaucoma	0 (0.00)	1 (20.00)	0 (0.00)	1 (14.29)	0.14	0 (0.00)	2 (7.69)	0.52
Cataract	0 (0.00)	1 (20.00)	1 (6.25)	1 (14.29)	0.41	0 (0.00)	3 (11.54)	0.28
Myopia	2 (14.29)	2 (40.00)	2 (12.50)	2 (28.57)	0.41	2 (12.50)	6 (23.08)	0.69
Ocular fundus disease	0 (0.00)	0 (0.00)	0 (0.00)	1 (14.29)	0.16	0 (0.00)	1 (3.85)	1.00
Ophthalmic medication (within one month)	2 (14.29)	2 (40.00)	2 (12.50)	2 (28.57)	0.48	2 (12.50)	6 (23.08)	0.69
Activity of ocular disease (within three months)	0 (0.00)	1 (20.00)	0 (0.00)	1 (14.29)	0.14	0 (0.00)	2 (7.69)	0.52
Ocular operation or trauma (within three months)	0 (0.00)	0 (0.00)	0 (0.00)	1 (14.29)	0.16	0 (0.00)	1 (3.85)	1.00
Typical symptoms								
Fever	9 (64.29)	5 (100.00)	13 (81.25)	6 (85.71)	0.43	11 (68.75)	22 (84.62)	0.27
Cough	9 (64.29)	5 (100.00)	11 (68.75)	3 (42.86)	0.26	12 (75%)	16 (61.54)	0.51
Pharyngodynia	4 (28.57)	0 (0.00)	2 (12.50)	0 (0.00)	0.33	4 (25%)	2 (7.69)	0.18
Anhelation	6 (42.86)	1 (20.00)	6 (37.50)	1 (14.29)	0.61	5 (31.25%)	9 (34.62)	1.00
Fatigue	6 (42.86)	2 (40.00)	4 (25.00)	4 (57.14)	0.48	6 (37.5%)	10 (38.46)	1.00
Muscle soreness	1 (7.14)	3 (60.00)	4 (25.00)	4 (57.14)	0.03	2 (1.25%)	10 (38.46)	0.09
Bosom frowsty and (or) ache	2 (14.29)	0 (0.00)	2 (12.50)	2 (28.57)	0.56	1 (6.25%)	5 (19.23)	0.38
Abdominal pain, diarrhea	1 (7.14)	0 (0.00)	3 (18.75)	1 (14.29)	0.84	1 (6.25%)	4 (15.38)	0.63

## Data Availability

All data generated or analyzed during this study are included in this published article.
